# Severity assessment of wheat stripe rust based on machine learning

**DOI:** 10.3389/fpls.2023.1150855

**Published:** 2023-03-17

**Authors:** Qian Jiang, Hongli Wang, Haiguang Wang

**Affiliations:** College of Plant Protection, China Agricultural University, Beijing, China

**Keywords:** wheat stripe rust, severity, disease assessment, image processing, machine learning, unsupervised learning, supervised learning

## Abstract

**Introduction:**

The accurate severity assessment of wheat stripe rust is the basis for the pathogen-host interaction phenotyping, disease prediction, and disease control measure making.

**Methods:**

To realize the rapid and accurate severity assessment of the disease, the severity assessment methods of the disease were investigated based on machine learning in this study. Based on the actual percentages of the lesion areas in the areas of the corresponding whole single diseased wheat leaves of each severity class of the disease, obtained after the image segmentation operations on the acquired single diseased wheat leaf images and the pixel statistics operations on the segmented images by using image processing software, under two conditions of considering healthy single wheat leaves or not, the training and testing sets were constructed by using two modeling ratios of 4:1 and 3:2, respectively. Then, based on the training sets, two unsupervised learning methods including *K*-means clustering algorithm and spectral clustering and three supervised learning methods including support vector machine, random forest, and *K*-nearest neighbor were used to build severity assessment models of the disease, respectively.

**Results:**

Regardless of whether the healthy wheat leaves were considered or not, when the modeling ratios were 4:1 and 3:2, satisfactory assessment performances on the training and testing sets can be achieved by using the optimal models based on unsupervised learning and those based on supervised learning. In particular, the assessment performances obtained by using the optimal random forest models were the best, with the accuracies, precisions, recalls, and F1 scores for all the severity classes of the training and testing sets equal to 100.00% and the overall accuracies of the training and testing sets equal to 100.00%.

**Discussion:**

The simple, rapid, and easy-to-operate severity assessment methods based on machine learning were provided for wheat stripe rust in this study. This study provides a basis for the automatic severity assessment of wheat stripe rust based on image processing technology, and provides a reference for the severity assessments of other plant diseases.

## 1 Introduction

Wheat stripe rust (wheat yellow rust), caused by *Puccinia striiformis* f. sp. *tritici* (*Pst*), is a devastating epidemic disease, which is widely distributed in wheat growing areas worldwide (Li and Zeng, 2002; Line, 2002; Chen, 2005; Wellings, 2011; Wang et al., 2014; Ali et al., 2017; Figueroa et al., 2018). As an air-borne fungal disease, wheat stripe rust can cause multiple uredinia on wheat leaves and a large number of *Pst* urediospores can be produced and released. The dispersal of *Pst* urediospores by wind can cause large-scale epidemics of the disease in wheat growing areas, which can seriously affect the yield and quality of wheat (Li and Zeng, 2002; Chen, 2005; Chen et al., 2014; Wang et al., 2014). In China, wheat stripe rust, one of the most important wheat diseases, has been a serious threat to wheat production safety because of its high epidemic frequency and severe destructiveness, and the pandemics of the disease have occurred many times, resulting in huge wheat yield losses (Li and Zeng, 2002; Chen et al., 2014; Wang et al., 2014; Liu et al., 2022). There are three epidemiological region systems of wheat stripe rust in China, including the northern China–north-western China–the middle and lower reaches of the Yangtze River epidemiological region system, Xinjiang epidemiological region system, and Yunnan epidemiological region system, and the disease cycles are completed by air dispersals of *Pst* urediospores in each epidemiological region system or across the epidemiological region systems (Li and Zeng, 2002; Chen et al., 2014; Wang et al., 2014). To effectively control the occurrence and epidemics of wheat stripe rust and to achieve sustainable management of the disease, it is of great significance to carry out surveys and monitoring of the disease.

Severity is a key indicator to describe the disease intensity of an investigated plant unit (a plant or a plant part such as leaf, fruit, or stem), and different severity levels of a plant disease are usually classified based on the ratios of the diseased areas of the investigated plant units to the areas of the corresponding whole investigated plant units (Nutter et al., 1991; Bock et al., 2022b). Disease severity assessment should be performed according to the severity grading standard of a plant disease to ensure the standardization and integrity of the obtained data. Severity is an important indicator to be determined in surveys and monitoring of wheat stripe rust. In China, the severity assessment of wheat stripe rust should be conducted according to the Rules for Monitoring and Forecast of the Wheat Stripe Rust (*Puccinia striiformis* West.) (National Standard of the People’s Republic China, GB/T 15795–2011). In this standard, a total of eight severity classes including 1%, 5%, 10%, 20%, 40%, 60%, 80%, and 100%, are classified based on the percentages of the lesion areas (Generally, the lesion area refers to the area covered by all the uredinia on a diseased wheat leaf.) in the areas of the corresponding whole single diseased wheat leaves. According to this severity grading standard, the disease intensity of a single diseased wheat leaf is treated as its nearest severity class based on the nearest-neighbor rule, and as the disease intensity is lower than the severity class of 1%, it is classified as the severity class of 1%.

At the present time, the severity assessments of wheat stripe rust are implemented mainly by using the visual observation method. This kind of artificial method requires assessors/raters with rich experience, and it is time-consuming and laborious. Most importantly, it is difficult to accurately estimate the percentages of the lesion areas in the areas of the corresponding whole single diseased wheat leaves according to the severity grading standard of wheat stripe rust (Jiang et al., 2022), thus it is difficult to obtain accurate severity assessment results by using this method. Because the investigation of disease incidence only needs to determine whether the wheat leaves are diseased or not and it is easier to accurately investigate or assess disease incidence than disease severity, it was reported that disease incidence could be used to estimate the severity of wheat stripe rust (Dong et al., 1990). However, the quantitative relationship between incidence and severity (*I*-*S* relationship) is affected by the values of disease incidences, the distribution of lesions on wheat plants, wheat resistance to *Pst*, and so on (Dong et al., 1990), leading to great limitations to the practical applications of the method by utilizing incidence to estimate severity.

The assessment of plant disease severity based on information technology has been paid more and more attention (Bock et al., 2010; Li et al., 2015; Barbedo, 2016; Mahlein, 2016; Bock et al., 2022a). The rapid development of information technology has promoted the applications of image processing technology (Bao et al., 2021; Jiang et al., 2021; Jiang et al., 2022), remote sensing technology (Huang et al., 2004; Wang et al., 2007; Zhao et al., 2014; Wang et al., 2016), and near infrared spectroscopy technology (Li et al., 2015) in severity assessment of wheat stripe rust. Nevertheless, the methods for severity assessment of wheat stripe rust based on remote sensing technology and near infrared spectroscopy technology are rarely used in practical disease surveys and monitoring due to the high prices of the required instruments and the need to further improve the applicability of the related methods in practical wheat production. Studies on severity assessment of wheat stripe rust based on image processing technology are increasing (Bao et al., 2021; Jiang et al., 2021; Jiang et al., 2022).

At present, there are two main methods based on image processing technology to be utilized to carry out severity assessment of wheat stripe rust. One method is to directly build the severity assessment models of wheat stripe rust based on the extracted features (e.g., color, shape, and texture features) from disease images and then to carry out disease severity assessment by using the built models (Bao et al., 2021). The other method is to use image processing technology to obtain the actual percentages of the lesion areas in the areas of the whole single diseased wheat leaves, then to directly compare the actual percentages to the percentages for the eight severity classes in the severity grading standard of wheat stripe rust, and to obtain the severity classes of the corresponding diseased wheat leaves finally (Jiang et al., 2021). However, the percentage of the lesion area in the area of a whole single diseased wheat leaf corresponding to a severity class in the severity grading standard of wheat stripe rust is not the actual percentage of the lesion area in the area of the whole single diseased wheat leaf, and importantly, there is a great difference between them. This has been verified by the studies conducted by Shang et al. (1990) and Jiang et al. (2022). By using a method based on the uredinium parameters in combination with actually measuring the amplified image of the selected wheat leaf with the most severe disease symptom in the field, Shang et al. (1990) determined actual coverage rates of the *Pst* uredinia corresponding to the severity classes of 1%, 5%, 10%, 20%, 40%, 60%, 80%, and 100%, which were 0.35%, 1.75%, 3.5%, 7%, 14%, 21%, 28%, and 35%, respectively. By using image processing software to perform the operations of image segmentation and pixel statistics based on the acquired single diseased wheat leaf images, Jiang et al. (2022) obtained the ranges of the actual percentages of the lesion areas in the areas of the corresponding whole single diseased wheat leaves for the severity classes of 1%, 5%, 10%, 20%, 40%, 60%, 80%, and 100%, which were [0.06%, 0.78%], [0.85%, 1.64%], [1.73%, 3.29%], [3.65%, 6.31%], [6.76%, 13.88%], [14.22%, 18.43%], [18.90%, 24.15%], and [24.54%, 36.49%], respectively. The severity assessment method by directly comparing the actual percentage of lesion area in the area of a whole single diseased wheat leaf to the percentages of lesion areas of the eight severity classes in the severity grading standard of wheat stripe rust, can lead to great errors in severity assessments, which will greatly influence the accurate severity assessments of the disease. Therefore, it is difficult to accurately assess the severity of wheat stripe rust by directly comparing the actual percentage of lesion area in the area of a whole single diseased wheat leaf to the percentages of lesion areas of the eight severity classes in the severity grading standard of the disease. To accurately carry out severity assessment of wheat stripe rust based on the actual percentages of lesion areas in the areas of the corresponding whole single diseased wheat leaves, Jiang et al. (2022) proposed two reference-range-based methods for severity assessment of wheat stripe rust, and satisfactory results with the assessment accuracies not lower than 85% were achieved by using the determined reference ranges to conduct severity assessments of the disease. However, the methods for severity assessment of wheat stripe rust proposed by Jiang et al. (2022) need to compare the actual percentages of lesion areas in the areas of the whole single diseased wheat leaves to the upper and lower limits of the determined reference range of the actual percentages of lesion areas for each severity class, and then the severity classes of the single diseased wheat leaves to be assessed can be determined accordingly.

To timely and accurately obtain the severity information of wheat stripe rust, it is necessary to develop a simple, rapid, accurate, and easy-to-operate severity assessment method for wheat stripe rust. On the basis of the study conducted by Jiang et al. (2022), the severity assessment methods based on machine learning were developed for wheat stripe rust in this study. The obtained actual percentages of the lesion areas in the areas of the corresponding whole single diseased wheat leaves were clustered into different severity classes by using two unsupervised learning methods including *K*-means clustering algorithm and spectral clustering, respectively, and the built clustering models were treated as the severity assessment models of wheat stripe rust based on unsupervised learning. Simultaneously, the severity assessment models of wheat stripe rust were built with the obtained actual percentage data by using three supervised learning methods including support vector machine (SVM), random forest (RF), and *K*-nearest neighbor (KNN), respectively. To ensure that healthy wheat leaves could be assessed, severity assessment models of wheat stripe rust were also built by using the above five modeling methods under the condition of considering single healthy wheat leaves. Finally, all the built models were used to carry out severity assessments of the single wheat leaves. The goal of this study is to overcome the difficulties in severity assessment of wheat stripe rust and to solve the problem of low assessment accuracy caused by directly comparing the actual percentages of lesion areas in the areas of the single diseased wheat leaves to the percentages of lesion areas of the eight severity classes in the severity grading standard of the disease. This study will provide rapid and accurate severity assessment methods for wheat stripe rust based on the actual percentages of the lesion areas in the areas of the corresponding whole single diseased wheat leaves, and also provide a reference and basis for the severity assessments of other plant diseases and the automatic assessments of plant disease severity.

## 2 Materials and methods

**Figure 1:**
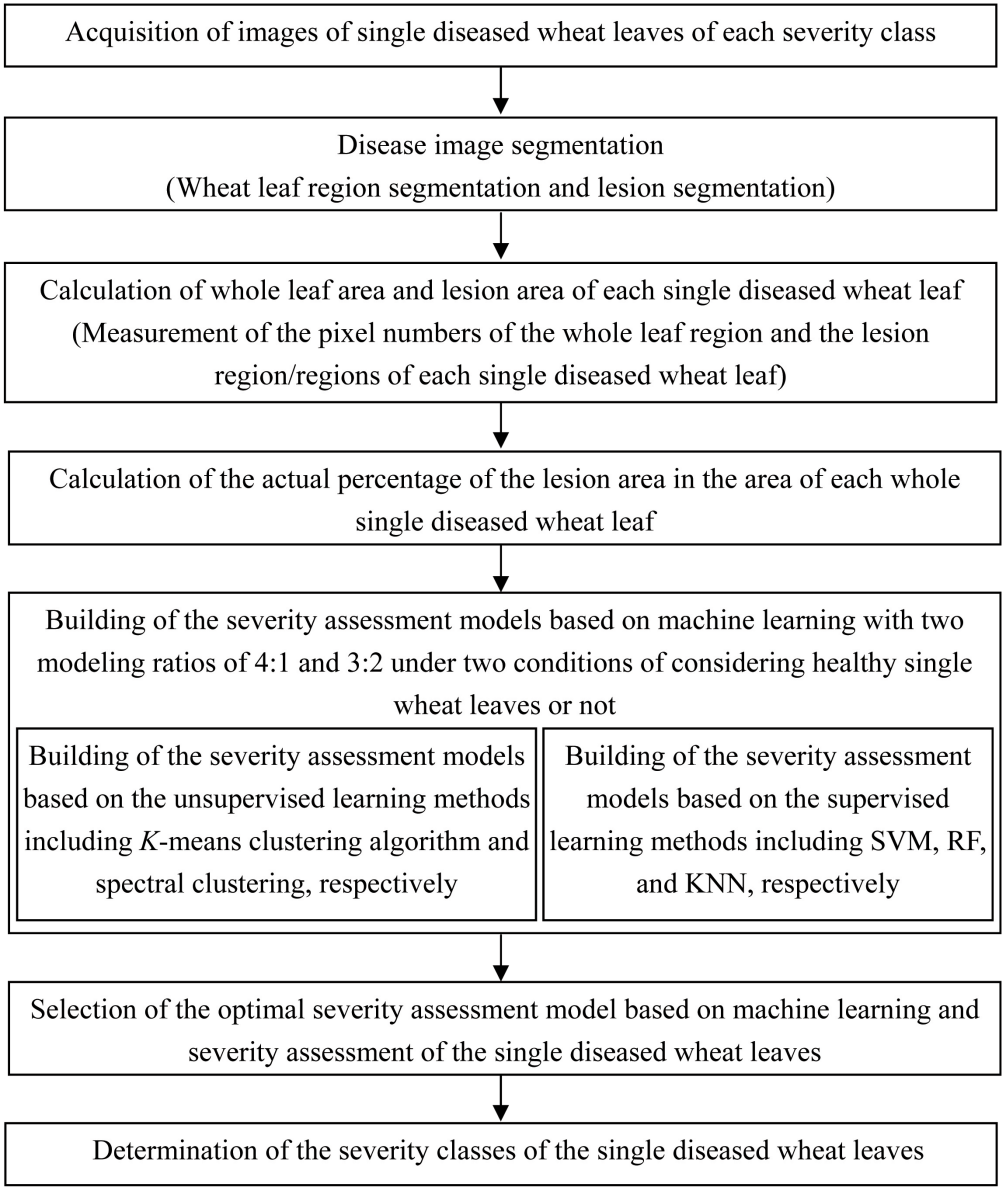
Work flow diagram for investigation of severity assessment methods of wheat stripe rust based on machine learning.

The main steps for developing the severity assessment methods of wheat stripe rust based on machine learning in this study are shown in **Figure 1**. Especially, to enable the built severity assessment models to be used for severity assessments of single wheat leaves including healthy leaves and to implement automatic disease assessments for acquired single wheat leaves in the future, in this study, the severity assessment of wheat stripe rust based on mechanical learning was investigated under two conditions of considering healthy single wheat leaves or not.

### 2.1 Disease images and image processing methods

The images of wheat stripe rust and the corresponding actual percentages of lesion areas in the areas of the single diseased wheat leaves used in this study were as same as those acquired by Jiang et al. (2022). Therefore, the images, data, and relevant methods used to obtain the images and data, are only briefly described here.

A total of 400 images of wheat stripe rust were used in this study, which were acquired by using 400 single diseased wheat leaves of eight severity classes of 1%, 5%, 10%, 20%, 40%, 60%, 80%, and 100% collected on the wheat plants in Shangzhuang Experimental Station of China Agricultural University, Beijing, China and an artificial climate chamber in the Laboratory of Macro-Phytopathology, China Agricultural University, Beijing, China, according to the Rules for Monitoring and Forecast of the Wheat Stripe Rust (*Puccinia striiformis* West.) as described above. For each severity class, 50 images were acquired (one image per single diseased wheat leaf) by using a Nikon D700 digital camera (Nikon Corp., Tokyo, Japan), a HUAWEI P30 smartphone, or an iPhone 6S smartphone. The sizes of the images acquired with the Nikon D700 digital camera, the HUAWEI P30 smartphone, and the iPhone 6S smartphone were 4256×2832, 3648×2736, and 4032×3024 pixels, respectively, and all the disease images were in the JEPG format. In the Adobe Photoshop 2022 software (Adobe Systems Incorporated, San Jose, CA, USA), leaf region segmentation and lesion region segmentation of the single diseased wheat leaf images were performed, and then the pixel number of the whole leaf region and the pixel number of the lesion region/regions for each single diseased wheat leaf were obtained by pixel statistics. Based on the pixel numbers of the whole leaf region and the lesion region/regions, the actual percentage of the lesion area in the area of the whole single diseased wheat leaf was calculated.

### 2.2 Building of the severity assessment models of wheat stripe rust based on machine learning under the condition without considering single healthy wheat leaves

The eight severity classes of wheat stripe rust were regarded as eight categories, i.e., the severity classes of 1%, 5%, 10%, 20%, 40%, 60%, 80%, and 100% were set to the severity categories of 1, 2, 3, 4, 5, 6, 7, and 8, respectively. Each category was composed of 50 single diseased wheat leaf specimens of wheat stripe rust. For each category, the actual percentages of the lesion areas in the corresponding whole leaf areas for the 50 specimens were sorted from large to small, and then, the specimens were sampled to construct the training set and the testing set by using the system sampling method with the modeling ratio of the number of specimens in the training set to the number of specimens in the testing set equal to 4:1 or 3:2. Subsequently, for each modeling ratio (4:1 or 3:2), the training sets of the eight categories (severity classes) were merged into new training set (Train840 or Train830) and the testing sets of the eight categories were merged into testing set (Test810 or Test820). For the modeling ratio of 4:1, the training set Train840 consisted of 320 specimens, and the corresponding testing set Test810 consisted of 80 specimens. For the modeling ratio of 3:2, the training set Train830 and the corresponding testing set Test820 consisted of 240 specimens and 160 specimens, respectively.

#### 2.2.1 Building of the severity assessment models of wheat stripe rust based on unsupervised learning under the condition without considering single healthy wheat leaves

The actual percentage of the lesion area in the whole wheat leaf area and the corresponding category number of each specimen in the training set Train840, the testing set Test810, the training set Train830, and the testing set Test820 were input into the data tables in Microsoft Excel 2016, and the corresponding data tables were named train840, test810, train830, and test820, respectively. In each data table, the data in the first column were the actual percentages of the lesion areas in the corresponding whole wheat leaf areas and were recorded as X; the data in the second column were the corresponding severity category numbers and were recorded as Y.

Under the condition without considering single healthy wheat leaves, based on the actual percentages of the lesion areas in the areas of the corresponding whole single diseased wheat leaves, the severity assessment models of wheat stripe rust were built with the two unsupervised learning methods including the *K*-means clustering algorithm and spectral clustering by using the programming language Python (version: 3.8.12) in the software Pycharm 2021.2.1, respectively.

The *K*-means clustering algorithm is an unsupervised learning clustering algorithm based on distance clustering. It separates the data samples into different categories by initializing centroids and constantly updating the clustering centroids, and the clustering will be accomplished until the data samples in each category does not change, the maximum number of iterations is reached, or the error is lower than the expected value (MacQueen, 1967). To build the severity assessment models of wheat stripe rust with the *K*-means clustering algorithm, firstly, the data X and Y were read from the data tables train840, test810, train830, and test820, respectively, by calling the read_excel() method from the Pandas library. Then, the reshape(-1,1) method was used to convert the data X from a one-dimensional array into a two-dimensional array with one column to meet the requirements of scikit-learn library (Pedregosa et al., 2011) for input data. Subsequently, the severity assessment models of wheat stripe rust were built with different parameters by calling the module sklearn.cluster from the scikit-learn library. The method fit() was used to train the models based on the data X of the training sets. The labels over the data X of a training set after clustering were viewed by using the labels_ attribute and were recorded as train_label, and the number of each label was counted by calling the value_counts() method from the Pandas library. The method predict() was used to predict the labels of the data X of the corresponding testing sets by using the trained models. The predicted labels over the data X of a testing set were recorded as test_label, and the number of each label was counted by calling the value_counts() method from the Pandas library. When the severity assessment model of wheat stripe rust was built with the *K*-means clustering algorithm based on the training set Train840, the parameter n_clusters, that is, the number of clusters to form (i.e., the number of centroids to generate), was set to 8; the parameter random_state, that is, the generator used to determine random number generation for centroid initialization, was set to 10; the parameter n_init, that is, the number of times for random initialization, was set to 1; the parameter init, that is, the method for initialization, was set to ‘random’, which means that the initial centroids were randomly selected from the training data; and the default values were used for the other parameters. When the severity assessment model of wheat stripe rust was built with the *K*-means clustering algorithm based on the training set Train830, the parameter n_clusters was set to 8, the parameter random_state was set to 8, the parameter n_init was set to 1, the parameter init was set to ‘random’, and the default values were used for the other parameters.

Spectral clustering is an unsupervised learning clustering algorithm based on graph theory, by firstly establishing the Laplacian matrix of the data of samples, then calculating the eigenvalues and eigenvectors of the matrix, subsequently constructing the eigenvector space, and finally, clustering the eigenvectors to accomplish the clustering of the data samples with a traditional clustering algorithm such as the *K*-means algorithm (Shi and Malik, 2000; von Luxburg, 2007). To build the severity assessment models of wheat stripe rust with spectral clustering, the data X and Y were firstly read from the data tables train840, test810, train830, and test820, respectively, by calling the method read_excel() from the Pandas library, and then, the reshape(-1,1) method was used to convert the data X from a one-dimensional array into a two-dimensional array with one column to meet the requirements of scikit-learn library (Pedregosa et al., 2011) for input data. Subsequently, by calling the module sklearn.cluster from the scikit-learn library, the severity assessment models of wheat stripe rust were built with spectral clustering with different parameters. The method fit() was used to train the models based on the data X of the training sets. The labels over the data X of a training set after clustering were found in the labels_ attribute and were recorded as train_label, and the number of each label was counted by using the method value_counts() of the Pandas library. The method fit_predict() was used to predict the labels of the data X of the corresponding testing sets by using the trained models. The predicted labels over the data X of a testing set were recorded as test_label, and the counts of the unique labels were obtained by using the method value_counts() of the Pandas library. To build the severity assessment model of wheat stripe rust with spectral clustering based on the training set Train840, the parameter n_clusters, that is, the number of clustering dimensions (the number of clusters to form), was set to 8; the parameter affinity, that is, the method used to construct the affinity matrix, was set to ‘nearest_neighbors’, which means that the affinity matrix was constructed by using the nearest neighbors method; the parameter n_neighbors, that is, the number of neighbors used to construct the affinity matrix by using the nearest neighbors method, was set to 10; the parameter assign_labels, that is, the strategy used to assign labels in the embedding space, was set to ‘discretize’, which means that discretization approach was used to assign labels in the embedding space; and the default values were used for the other parameters. To build the severity assessment model of wheat stripe rust with spectral clustering based on the training set Train830, the parameter n_clusters was set to 8, the parameter affinity was set to ‘nearest_neighbors’, the parameter n_neighbors was set to 7, the parameter assign_labels was set to ‘discretize’, and the default values were used for the other parameters.

#### 2.2.2 Building of the severity assessment models of wheat stripe rust based on supervised learning under the condition without considering single healthy wheat leaves

Under the condition without considering single healthy wheat leaves, based on the actual percentages of the lesion areas in the areas of the corresponding whole single diseased wheat leaves, the severity assessment models of wheat stripe rust were built with the three supervised learning methods including SVM (Cortes and Vapnik, 1995), RF (Breiman, 2001), and KNN (Cover and Hart, 1967), respectively.

By using the C-SVM in the LIBSVM-3.23 package developed by Chih-Jen Lin Group from National Taiwan University, Taiwan, China (Chang and Lin, 2011), the severity assessment SVM models of wheat stripe rust were built in this study. When building a SVM model for severity assessment of wheat stripe rust, the RBF kernel function was used. The grid search algorithm was utilized to determine the optimal values for both penalty parameter *C* and kernel function parameter *g* by searching in a range of 2^-10^ – 2^10^ with the searching step equal to 0.4. By using the 3-fold cross-validation method, when the assessment accuracy was the highest for the training set, the corresponding values of the parameters *C* and *g* were regarded as the optimal parameters to build the optimal SVM model for severity assessment of wheat stripe rust.

Building of the severity assessment RF models of wheat stripe rust was implemented in the software MATLAB R2019b (MathWorks, Natick, MA, USA). When building the severity assessment RF models of wheat stripe rust based on a training set, the number of decision trees, a key parameter for modeling, was set to 10, 20, 30, 40, 50, 60, 70, 80, 90, and 100, respectively, and the other parameters with the default values were used. The model performance for severity assessment of wheat stripe rust was used to determine the optimal number of decision trees for modeling. Then, the determined optimal number of decision trees and the other parameters with the default values were used to build the optimal RF model for severity assessment of wheat stripe rust.

Building of the severity assessment KNN models of wheat stripe rust was implemented by using the KNN classifier in the software MATLAB R2019b. When building the severity assessment KNN models of wheat stripe rust based on a training set, Euclidean distance was selected as the default distance metric measure, the values of the key parameter *K* were selected in a range of 1–20 with the searching step of 2, and the other parameters with the default values were used. According to the assessment accuracies of the models, the optimal value of *K* was determined when the assessment accuracy was the highest for the training set. Then, the optimal KNN model for severity assessment of wheat stripe rust was built with the optimal *K* and the other parameters with the default values.

### 2.3 Building of the severity assessment models of wheat stripe rust based on machine learning under the condition of considering single healthy wheat leaves

To ensure that the built severity assessment models of wheat stripe rust could be used to assess the specimens of healthy wheat leaves, the actual percentage of the lesion area to the whole leaf area of a single healthy wheat leaf was set to 0% (i.e., the corresponding severity class was set to 0%.), and 50 ‘0%’ for healthy wheat leaves were added and then were numbered, respectively. The severity category of each healthy wheat leaf was set to 0, and the severity categories of the severity classes of 1%, 5%, 10%, 20%, 40%, 60%, 80%, and 100% were set to 1, 2, 3, 4, 5, 6, 7, and 8, respectively. Thus, the severity classes of wheat stripe rust were regarded as nine categories (0–8) and each category consisted of 50 single wheat leaf specimens. By using the method as described above, the actual percentages of the lesion areas in the corresponding whole leaf areas for the 50 specimens of each category were sorted from large to small, and then the specimens were sampled to construct the training set and the testing set by using the system sampling method with the modeling ratio of the number of specimens in the training set to the number of specimens in the testing set equal to 4:1 or 3:2. Subsequently, the training sets of the nine categories were merged into new training set Train940 or Train930, and the testing sets of the nine categories were merged into the corresponding new testing set Test910 or Test920. For the modeling ratio of 4:1, the training set Train940 and the corresponding testing set Test910 consisted of 360 specimens and 90 specimens, respectively. For the modeling ratio of 3:2, the training set Train930 and the corresponding testing set Test920 consisted of 270 specimens and 180 specimens, respectively.

#### 2.3.1 Building of the severity assessment models of wheat stripe rust based on unsupervised learning under the condition of considering single healthy wheat leaves

By using the same method as described above, the data tables train940, test910, train930, and test920 were created based on the training set Train940, the testing set Test910, the training set Train930, and the testing set Test920, respectively. In each data table, the data in the first column recorded as X were the actual percentages of the lesion areas in the corresponding whole wheat leaf areas and that in the second column recorded as Y were the corresponding category numbers of disease severity.

By using the similar methods to build the severity assessment models of wheat stripe rust with the two unsupervised learning methods under the condition without considering single healthy wheat leaves as described above, the severity assessment models of wheat stripe rust under the condition of considering single healthy wheat leaves were built with the two unsupervised learning methods including the *K*-means clustering algorithm and spectral clustering, respectively, based on the actual percentages of the lesion areas in the areas of the corresponding whole single wheat leaves by using the programming language Python (version: 3.8.12) in the software Pycharm 2021.2.1. To build the severity assessment model of wheat stripe rust with the *K*-means clustering algorithm based on the training set Train940, the parameter n_clusters was set to 9, the parameter random_state was set to 8, the parameter n_init was set to 1, the parameter init was set to ‘random’, and the default values were used for the other parameters. To build the severity assessment model of wheat stripe rust with the *K*-means clustering algorithm based on the training set Train930, the parameter n_clusters was set to 9, the parameter random_state was set to 7, the parameter n_init was set to 1, the parameter init was set to ‘random’, and the default values were used for the other parameters. To build the severity assessment model of wheat stripe rust with spectral clustering based on the training set Train940, the parameter n_clusters was set to 9, the parameter affinity was set to ‘nearest_neighbors’, the parameter n_neighbors was set to 10, the parameter assign_labels was set to ‘discretize’, and the default values were used for the other parameters. To build the severity assessment model of wheat stripe rust with spectral clustering based on the training set Train930, the parameter n_clusters was set to 9, the parameter affinity was set to ‘nearest_neighbors’, the parameter n_neighbors was set to 8, the parameter assign_labels was set to ‘discretize’, and the default values were used for the other parameters.

#### 2.3.2 Building of the severity assessment models of wheat stripe rust based on supervised learning under the condition of considering single healthy wheat leaves

By using the same methods to build the severity assessment models of wheat stripe rust with the three supervised learning methods under the condition without considering single healthy wheat leaves as described above, the severity assessment models of wheat stripe rust under the condition of considering single healthy wheat leaves were built based on the actual percentages of the lesion areas in the areas of the corresponding whole single wheat leaves with the three supervised learning methods including SVM, RF, and KNN, respectively.

### 2.4 Performance evaluation of the built severity assessment models of wheat stripe rust

By using the severity assessment models of wheat stripe rust built with the two unsupervised learning methods (including the *K*-means clustering algorithm and spectral clustering) and the three supervised learning methods (including the SVM, RF, and KNN), the severity assessments of the corresponding training sets and testing sets were carried out. The accuracy, precision, recall, and F1 score of the severity assessments for the specimens of each severity class in the corresponding training sets and testing sets and the overall accuracies of the corresponding training sets and testing sets were calculated, respectively, aiming to evaluate the assessment performances of the built models and to choose the optimal severity assessment model of wheat stripe rust for each modeling ratio under the condition without considering single healthy wheat leaves or under the condition of considering single healthy wheat leaves. The accuracy for a severity class of wheat stripe rust is the percentage of the number of the correctly assessed single leaf specimens in the total number of single leaf specimens to be assessed. The precision is the percentage of the number of actual single leaf specimens at a severity class of wheat stripe rust in the number of the single leaf specimens assessed as the severity class. Recall is the percentage of the number of the single leaf specimens assessed as a severity class of wheat stripe rust in the number of the single leaf specimens actually at the severity class. F1 score is the harmonic mean of precision and recall. The overall accuracy is the percentage of the number of the single leaf specimens that are correctly assessed as the corresponding severity classes in the total number of single leaf specimens to be assessed. All the five evaluation indicators of severity assessment were calculated according to Formulas (1)–(5), respectively.


(1) $$\text{Accuracy}=\frac{\text{TP}+\text{TN}}{\text{TP}+\text{TN}+\text{FP}+\text{FN}}\times 100\%$$

(2) $$\text{Precision}=\frac{\text{TP}}{\text{TP}+\text{FP}}\times 100\%$$

(3) $$\text{Recall}=\frac{\text{TP}}{\text{TP}+\text{FN}}\times 100\%$$

(4) $$F1 score=\frac{2\times \text{Precision}\times \text{Recall}}{\text{Precision}+\text{Recall}}$$

(5) $$\text{Overall accuracy}=\frac{\sum_{i=1}^{\left|N\right|}{\text{TP}}_{i}}{\sum_{i=1}^{\left|N\right|}\left({\text{TP}}_{i}+{\text{FN}}_{i}\right)}\times 100\%$$

where TP (true positive) is the number of the single leaf specimens actually at a severity class assessed as the severity class; FN (false negative) is the number of the single leaf specimens actually at the severity class assessed as the other severity classes; FP (false positive) is the number of the single leaf specimens actually at other severity classes assessed as the severity class; TN (true positive) is the number of the single leaf specimens actually at other severity classes correctly assessed as the corresponding other severity classes; *N* is the number of severity classes; and *i* is the *i*th severity class.

## 3 Results

### 3.1 Statistical results of the actual percentage data of the lesion areas in the areas of the corresponding whole single diseased wheat leaves at each severity class of wheat stripe rust

Statistical analysis of the data of the actual percentages of the lesion areas in the areas of the 50 corresponding whole single diseased wheat leaves at each severity class of wheat stripe rust, was conducted by using the UNIVARIATE procedure in the software SAS 9.4 (SAS Institute Inc. Cary, NC, USA). The minimum, maximum, mean, and standard deviation of the actual percentages of the lesion areas corresponding to each severity class of wheat stripe rust were obtained, as shown in **Table 1**. For the actual percentages of the lesion areas corresponding to the severity classes of 1%, 5%, 10%, 20%, 40%, 60%, 80%, and 100%, the minimum values were 0.06%, 0.85%, 1.73%, 3.65%, 6.76%, 14.22%, 18.90%, and 24.54%, respectively; the maximum values were 0.78%, 1.64%, 3.29%, 6.31%, 13.88%, 18.43%, 24.15%, and 36.49%, respectively; and the means were 0.40%, 1.27%, 2.50%, 4.92%, 9.88%, 16.61%, 21.24%, and 30.53%, respectively. The results showed that the actual percentages of the lesion areas corresponding to each severity class of wheat stripe rust were obviously lower than the percentage of the lesion area for the corresponding severity class in the severity grading standard of wheat stripe rust as described above.

**Table 1:** Statistics of the actual percentage data of the lesion areas in the areas of the whole diseased wheat leaves for the 50 acquired specimens corresponding to each severity class of wheat stripe rust including the minimum, maximum, mean, and standard deviation.

Severity class	Minimum of the actual percentages of the lesion areas	Maximum of the actual percentages of the lesion areas	Mean of the actual percentages of the lesion areas	Standard deviation of the actual percentages of the lesion areas
1%	0.06%	0.78%	0.40%	0.18%
5%	0.85%	1.64%	1.27%	0.23%
10%	1.73%	3.29%	2.50%	0.42%
20%	3.65%	6.31%	4.92%	0.77%
40%	6.76%	13.88%	9.88%	1.95%
60%	14.22%	18.43%	16.61%	1.20%
80%	18.90%	24.15%	21.24%	1.41%
100%	24.54%	36.49%	30.53%	3.17%

### 3.2 Severity assessment results obtained by using the severity assessment models of wheat stripe rust built based on the two unsupervised learning methods including the *k*-means clustering algorithm and spectral clustering under the condition without considering single healthy wheat leaves

Under the condition without considering single healthy wheat leaves, by using the severity assessment models of wheat stripe rust built based on the two unsupervised learning methods including the *K*-means clustering algorithm and spectral clustering, the severity assessments of the specimens of all the severity classes in the training and testing sets were carried out. The severity assessment results obtained by using the two unsupervised learning methods are shown in **Tables 2**, **3**, respectively.

**Table 2:** Severity assessment results of the single diseased wheat leaves with the actual percentages of lesion areas of all the severity classes of wheat stripe rust contained in the training and testing sets, obtained by using the severity assessment models of wheat stripe rust built based on the *K*-means clustering algorithm under the condition without considering single healthy wheat leaves.

Dataset	Severity category	Severity class	Accuracy	Precision	Recall	F1 score	Overall accuracy
Train840	1	1%	99.38%	95.24%	100.00%	97.56%	87.81%
	2	5%	96.25%	79.17%	95.00%	86.36%	
	3	10%	93.13%	71.43%	75.00%	73.17%	
	4	20%	93.75%	77.78%	70.00%	73.68%	
	5	40%	96.88%	100.00%	75.00%	85.71%	
	6	60%	99.06%	93.02%	100.00%	96.39%	
	7	80%	98.44%	90.70%	97.50%	93.98%	
	8	100%	98.75%	100.00%	90.00%	94.74%	
Test810	1	1%	98.75%	90.91%	100.00%	95.24%	87.50%
	2	5%	95.00%	75.00%	90.00%	81.82%	
	3	10%	92.50%	70.00%	70.00%	70.00%	
	4	20%	93.75%	77.78%	70.00%	73.68%	
	5	40%	97.50%	100.00%	80.00%	88.89%	
	6	60%	100.00%	100.00%	100.00%	100.00%	
	7	80%	98.75%	90.91%	100.00%	95.24%	
	8	100%	98.75%	100.00%	90.00%	94.74%	
Train830	1	1%	99.17%	93.75%	100.00%	96.77%	83.75%
	2	5%	95.83%	77.78%	93.33%	84.85%	
	3	10%	92.50%	68.75%	73.33%	70.97%	
	4	20%	93.33%	76.92%	66.67%	71.43%	
	5	40%	97.08%	100.00%	76.67%	86.79%	
	6	60%	96.67%	78.95%	100.00%	88.24%	
	7	80%	95.00%	82.14%	76.67%	79.31%	
	8	100%	97.92%	100.00%	83.33%	90.91%	
Test820	1	1%	98.75%	90.91%	100.00%	95.24%	83.75%
	2	5%	95.00%	75.00%	90.00%	81.82%	
	3	10%	92.50%	70.00%	70.00%	70.00%	
	4	20%	93.75%	77.78%	70.00%	73.68%	
	5	40%	96.88%	100.00%	75.00%	85.71%	
	6	60%	96.88%	80.00%	100.00%	88.89%	
	7	80%	95.63%	84.21%	80.00%	82.05%	
	8	100%	98.13%	100.00%	85.00%	91.89%	

**Table 3:** Severity assessment results of the single diseased wheat leaves with the actual percentages of lesion areas of all the severity classes of wheat stripe rust contained in the training and testing sets, obtained by using the severity assessment models of wheat stripe rust built based on spectral clustering under the condition without considering single healthy wheat leaves.

Dataset	Severity category	Severity class	Accuracy	Precision	Recall	F1 score	Overall accuracy
Train840	1	1%	100.00%	100.00%	100.00%	100.00%	97.50%
	2	5%	98.44%	88.89%	100.00%	94.12%	
	3	10%	98.44%	100.00%	87.50%	93.33%	
	4	20%	100.00%	100.00%	100.00%	100.00%	
	5	40%	100.00%	100.00%	100.00%	100.00%	
	6	60%	100.00%	100.00%	100.00%	100.00%	
	7	80%	99.06%	93.02%	100.00%	96.39%	
	8	100%	99.06%	100.00%	92.50%	96.10%	
Test810	1	1%	100.00%	100.00%	100.00%	100.00%	98.75%
	2	5%	98.75%	90.91%	100.00%	95.24%	
	3	10%	98.75%	100.00%	90.00%	94.74%	
	4	20%	100.00%	100.00%	100.00%	100.00%	
	5	40%	100.00%	100.00%	100.00%	100.00%	
	6	60%	100.00%	100.00%	100.00%	100.00%	
	7	80%	100.00%	100.00%	100.00%	100.00%	
	8	100%	100.00%	100.00%	100.00%	100.00%	
Train830	1	1%	100.00%	100.00%	100.00%	100.00%	97.50%
	2	5%	98.75%	90.91%	100.00%	95.24%	
	3	10%	98.75%	100.00%	90.00%	94.74%	
	4	20%	100.00%	100.00%	100.00%	100.00%	
	5	40%	100.00%	100.00%	100.00%	100.00%	
	6	60%	100.00%	100.00%	100.00%	100.00%	
	7	80%	98.75%	90.91%	100.00%	95.24%	
	8	100%	98.75%	100.00%	90.00%	94.74%	
Test820	1	1%	100.00%	100.00%	100.00%	100.00%	96.25%
	2	5%	98.13%	86.96%	100.00%	93.02%	
	3	10%	98.13%	100.00%	85.00%	91.89%	
	4	20%	100.00%	100.00%	100.00%	100.00%	
	5	40%	99.38%	100.00%	95.00%	97.44%	
	6	60%	99.38%	95.24%	100.00%	97.56%	
	7	80%	98.75%	90.91%	100.00%	95.24%	
	8	100%	98.75%	100.00%	90.00%	94.74%	

Under the condition without considering single healthy wheat leaves, when the modeling ratio was 4:1, the severity assessment model of wheat stripe rust was built by using the *K*-means clustering algorithm based on the training set Train840. By using the built model to conduct the severity assessments of the specimens in the training set Train840, the obtained results, as shown in **Table 2**, demonstrated that, for all the severity classes of wheat stripe rust, the lowest accuracy of 93.13% and the highest accuracy of 99.38% were obtained; the lowest and highest precisions of 71.43% and 100.00% were obtained, respectively; the lowest recall was 70.00%, and the highest recall was 100.00%; and the lowest F1 score was 73.17%, and the highest F1 score was 97.56%. By using the built model to conduct the severity assessments of the specimens in the testing set Test810, the results showed that, for all the severity classes of wheat stripe rust, the lowest and highest accuracies were 92.50% and 100.00%, respectively; the lowest values for precision, recall, and F1 score were all 70.00%, and the highest values for precision, recall, and F1 score were all 100.00%. There were large differences between the severity assessment results for each severity class in the training set Train840 or the testing set Test810 obtained by using the model built based on the *K*-means clustering algorithm. In detail, for all the severity classes, the obtained accuracies were very high; however, there were large differences in precision, recall, and F1 score. For the built severity assessment model, the overall accuracy of the training set Train840 was 87.81%, and that of the corresponding testing set Test810 was 87.50%.

Under the condition without considering single healthy wheat leaves, when the modeling ratio was 4:1, the severity assessment model of wheat stripe rust was built based on the training set Train840 by using spectral clustering. As shown in **Table 3**, the results obtained by using the built model to conduct the severity assessments of the specimens in the training set Train840 demonstrated that, for all the severity classes of wheat stripe rust, the lowest accuracy was 98.44% and the highest accuracy was 100.00%; except that the precisions for the severity classes of 5% and 80% were 88.89% and 93.02%, respectively, the precisions for all other severity classes were 100.00%; except that the recalls for the severity classes of 10% and 100% were 87.50% and 92.50%, respectively, the recalls for all other severity classes were 100.00%; and the lowest F1 score was 93.33%, and the highest F1 score was 100.00%. By using the built model to conduct the severity assessments of the specimens in the testing set Test810, the results showed that, except that the accuracies for the severity classes of 5% and 10% were both 98.75%, the accuracies for all other severity classes were 100.00%; except that the precision for the severity class of 5% was 90.91%, the precisions for all other severity classes were 100.00%; except that the recall for the severity class of 10% was 90.00%, the recalls for all other severity classes were 100.00%; except that the F1 scores for the severity classes of 5% and 10% were 95.24% and 94.74%, respectively, the F1 scores for all other severity classes were 100.00%. For the severity assessment model of wheat stripe rust built based on the training set Train840 by using spectral clustering, the overall accuracies of the training set used for modeling and the corresponding testing set Test810 were 97.50% and 98.75%, respectively. The results indicated that very good severity assessment results of the training and testing sets were achieved by using the severity assessment model built based on spectral clustering, when the modeling ratio was 4:1 under the condition without considering single healthy wheat leaves. There were relatively small differences among the severity assessment performances of the built model on all the severity classes of wheat stripe rust in the training set Train840 or the testing set Test810.

Under the condition without considering single healthy wheat leaves, when the modeling ratio was 3:2, by using the severity assessment model of wheat stripe rust built with the *K*-means clustering algorithm based on the training set Train830, the severity assessments of the specimens in the training set Train830 and the corresponding testing set Test820 were implemented. As shown in **Table 2**, for all the severity classes of wheat stripe rust in the training set Train830, the lowest accuracy was 92.50% and the highest accuracy was 99.17%; the lowest precision was 68.75% and the highest precision was 100.00%; the lowest recall was 66.67%, and the highest recall was 100.00%; and the lowest F1 score was 70.97%, and the highest F1 score was 96.77%. The results showed that, for all the severity classes of wheat stripe rust in the testing set Test820, the lowest and highest accuracies were 92.50% and 98.75%, respectively; the lowest and highest precisions were 70.00% and 100.00%, respectively; the lowest and highest recalls were 70.00% and 100.00%, respectively; and the lowest and highest F1 scores were 70.00% and 95.24%, respectively. In terms of precision, recall, and F1 score, there were great differences between the severity assessment results for each severity class in the training set Train830 or the testing set Test820 achieved by using the model built based on the *K*-means clustering algorithm under the condition without considering single healthy wheat leaves. For the built severity assessment model, the overall accuracies of the training set Train830 and the corresponding testing set Test820 were both 83.75%.

Under the condition without considering single healthy wheat leaves, when the modeling ratio was 3:2, the severity assessment model of wheat stripe rust built based on the training set Train830 by using spectral clustering was used to carry out the severity assessments of the specimens in the training set Train830 and the corresponding testing set Test820. As shown in **Table 3**, for the training set Train830, except that the accuracies for the severity classes of 5%, 10%, 80%, and 100% were all 98.75%, the accuracies for all other severity classes were 100.00%; except that the precisions for the severity classes of 5% and 80% were both 90.91%, the precisions for all other severity classes were 100.00%; except that the recalls for the severity classes of 10% and 100% were both 90.00%, the recalls for all other severity classes were 100.00%; and among the F1 scores for all the severity classes, the lowest and highest F1 values were 94.74% and 100.00%, respectively. The results showed that, for all the severity classes of wheat stripe rust in the testing set Test820, the lowest and highest accuracies were 98.13% and 100.00%, respectively; the lowest and highest precisions were 86.96% and 100.00%, respectively; except that the recalls for the severity classes of 10%, 40%, and 100% were 85.00%, 95.00%, and 90.00%, respectively, the recalls for other severity classes were all 100.00%; and the lowest and highest F1 scores were 91.89% and 100.00%, respectively. For the built severity assessment model of wheat stripe rust based on spectral clustering, the overall accuracies of the training set Train830 and the corresponding testing set Test820 were 97.50% and 96.25%, respectively. The results indicated that, under the condition without considering single healthy wheat leaves, very good severity assessment results of the training and testing sets by using the severity assessment model built based on spectral clustering when the modeling ratio was 3:2, were achieved, and there were relatively small differences among the severity assessment performances of the built model on all the severity classes in the training set Train830 used for modeling or the corresponding testing set Test820.

The results indicated that, when the severity assessments of the single diseased leaf image datasets of wheat stripe rust without single healthy wheat leaves were conducted by using the severity assessment model built based on the two unsupervised learning methods including the *K*-means clustering algorithm and spectral clustering, respectively, the acceptable assessment results could be achieved. Under the two conditions with the modeling ratios equal to 4:1 and 3:2, there was little difference between the assessment performances obtained by using the severity assessment models of wheat stripe rust built based on each of the two unsupervised learning methods. However, the assessment performance obtained by using the severity assessment model built when the modeling ratio was 4:1, was slightly better than that obtained by using the severity assessment model built when the modeling ratio was 3:2. In the case of any modeling ratio, the assessment performance obtained by using the severity assessment model of wheat stripe rust based on spectral clustering was better than that obtained by using the severity assessment model of wheat stripe rust based on the *K*-means clustering algorithm, and the severity assessment model of wheat stripe rust based on spectral clustering could achieve very ideal assessment results on both the training set and the corresponding testing set, indicating that the severity assessment model of wheat stripe rust based on spectral clustering could be used as the optimal model for severity assessment of wheat stripe rust. Therefore, to achieve ideal severity assessment results by using the severity assessment method of wheat stripe rust based on unsupervised learning, the spectral clustering method can be used to build the severity assessment model of the disease. The results indicated that the wheat stripe rust severity assessment methods based on unsupervised learning proposed in this study could be applied to severity assessment of the disease under the condition without considering single healthy wheat leaves.

### 3.3 Severity assessment results obtained by using the severity assessment models of wheat stripe rust built based on the three supervised learning methods including the SVM, RF, and KNN under the condition without considering single healthy wheat leaves

Under the condition without considering single healthy wheat leaves, when the modeling ratio was 4:1, based on the training set Train840, the optimal SVM model for severity assessment of wheat stripe rust was built with the optimal parameter *C* equal to 1.741 and the optimal parameter *g* equal to 6.964; the optimal RF model for severity assessment of wheat stripe rust was built with the optimal number of decision trees equal to 10; and the optimal KNN model for severity assessment of wheat stripe rust was built with the optimal *K* of 3. Under the condition without considering single healthy wheat leaves, when the modeling ratio was 3:2, based on the training set Train830, the optimal SVM model for severity assessment of wheat stripe rust was built with the optimal parameter *C* of 0.758 and the optimal parameter *g* of 6.964; the optimal RF model for severity assessment of wheat stripe rust was built with the optimal number of decision trees equal to 20; and the optimal KNN model for severity assessment of wheat stripe rust was built with the optimal *K* equal to 5.

Under the condition without considering single healthy wheat leaves, when the modeling ratios were 4:1 and 3:2, the optimal SVM models and the optimal RF models built for severity assessment of wheat stripe rust were used to carry out the severity assessments of the specimens in the training sets (Train840 and Train830) and the testing sets (Test810 and Test820). For all the severity classes of wheat stripe rust in the training sets (Train840 and Train830) and the testing sets (Test810 and Test820), the accuracies, precisions, recalls, and F1 scores were all 100.00%. For each modeling ratio, by using the optimal SVM model and the optimal RF model built for severity assessment of wheat stripe rust, the overall accuracies of the training set that was used for modeling and the corresponding testing set were both 100.00% (**Table 4**).

**Table 4:** Overall accuracies of the training and testing sets obtained by using the built severity assessment SVM, RF, and KNN models of wheat stripe rust under the condition without considering single healthy wheat leaves.

Modeling method	Modeling ratio	Overall accuracy of the training set	Overall accuracy of the testing set
SVM	4:1	100.00%	100.00%
	3:2	100.00%	100.00%
RF	4:1	100.00%	100.00%
	3:2	100.00%	100.00%
KNN	4:1	99.69%	100.00%
	3:2	100.00%	100.00%

The table shows only the assessment results of the optimal severity assessment SVM, RF, and KNN models of wheat stripe rust.

Under the condition without considering single healthy wheat leaves, when the modeling ratio were 4:1, by using the optimal KNN model to perform the severity assessments of the specimens in the training set Train840 for modeling and the corresponding testing set Test810, the accuracy, precision, recall, and F1 score for the severity class of 1% in the training set Train840 were 99.69%, 100.00%, 97.50%, and 98.73%, respectively; the accuracy, precision, recall, and F1 score for the severity class of 5% in the training set Train840 were 99.69%, 97.56%, 100.00%, and 98.77%, respectively; and the accuracies, precisions, recalls, and F1 scores for all other severity classes in the training set Train840 and all the severity classes in the testing set Test810 were 100.00%. Under the condition without considering single healthy wheat leaves, when the modeling ratios was 3:2, by using the optimal KNN model built based on the training set Train830 for severity assessment of wheat stripe rust, the accuracies, precisions, recalls, and F1 scores were all 100.00% for all the severity classes of wheat stripe rust in the training set Train830 and the testing set Test820. As shown in **Table 4**, for the modeling ratio of 4:1, by using the optimal KNN model built for severity assessment of wheat stripe rust based on the training set Train840, the overall accuracies of the training set Train840 and the corresponding testing set Test810 were 99.69% and 100.00%, respectively. For the modeling ratio of 3:2, by using the optimal KNN model built for severity assessment of wheat stripe rust based on the training set Train830, the overall accuracies of the training set Train830 and the corresponding testing set Test820 were both 100.00%.

The results showed that, under the condition without considering single healthy wheat leaves, by using the optimal SVM, RF, and KNN models for severity assessment of wheat stripe rust, very good severity assessment performances on the training sets (Train840 and Train830) and testing sets (Test810 and Test820) were achieved. In comparison, in the case of the two modeling ratios of 4:1 and 3:2, among the three kinds of models, the severity assessment models of wheat stripe rust built based on SVM and RF were optimal, and the corresponding overall accuracies of the training sets and the testing sets reached 100.00%. The results indicated that the methods for severity assessment of wheat stripe rust based on supervised learning proposed in this study could be applied to severity assessment of the disease under the condition without considering single healthy wheat leaves.

### 3.4 Severity assessment results obtained by using the severity assessment models of wheat stripe rust built based on the two unsupervised learning methods including the *K*-means clustering algorithm and spectral clustering under the condition of considering single healthy wheat leaves

Under the condition of considering single healthy wheat leaves, the severity assessment models of wheat stripe rust, built based on the two unsupervised learning methods including the *K*-means clustering algorithm and spectral clustering, were used to perform the severity assessments of the specimens of all the severity classes in the training and testing sets. The severity assessment results obtained by using the two unsupervised learning methods are shown in **Tables 5**, **6**, respectively.

**Table 5:** Severity assessment results of the single wheat leaves with the actual percentages of lesion areas of all the severity classes of wheat stripe rust contained in the training and testing sets, obtained by using the severity assessment models of wheat stripe rust built based on the *K*-means clustering algorithm under the condition of considering single healthy wheat leaves.

Dataset	Severity category	Severity class	Accuracy	Precision	Recall	F1 score	Overall accuracy
Train940	0	0%	96.67%	76.92%	100.00%	86.96%	84.72%
	1	1%	95.00%	82.35%	70.00%	75.68%	
	2	5%	95.56%	77.27%	85.00%	80.95%	
	3	10%	93.89%	71.43%	75.00%	73.17%	
	4	20%	94.44%	77.78%	70.00%	73.68%	
	5	40%	97.22%	100.00%	75.00%	85.71%	
	6	60%	99.17%	93.02%	100.00%	96.39%	
	7	80%	98.61%	90.70%	97.50%	93.98%	
	8	100%	98.89%	100.00%	90.00%	94.74%	
Test910	0	0%	96.67%	76.92%	100.00%	86.96%	85.56%
	1	1%	95.56%	87.50%	70.00%	77.78%	
	2	5%	95.56%	75.00%	90.00%	81.82%	
	3	10%	93.33%	70.00%	70.00%	70.00%	
	4	20%	94.44%	77.78%	70.00%	73.68%	
	5	40%	97.78%	100.00%	80.00%	88.89%	
	6	60%	100.00%	100.00%	100.00%	100.00%	
	7	80%	98.89%	90.91%	100.00%	95.24%	
	8	100%	98.89%	100.00%	90.00%	94.74%	
Train930	0	0%	96.67%	76.92%	100.00%	86.96%	85.19%
	1	1%	95.56%	87.50%	70.00%	77.78%	
	2	5%	95.93%	77.14%	90.00%	83.08%	
	3	10%	93.70%	70.97%	73.33%	72.13%	
	4	20%	94.44%	77.78%	70.00%	73.68%	
	5	40%	97.41%	100.00%	76.67%	86.79%	
	6	60%	99.26%	93.75%	100.00%	96.77%	
	7	80%	98.52%	90.63%	96.67%	93.55%	
	8	100%	98.89%	100.00%	90.00%	94.74%	
Test920	0	0%	96.67%	76.92%	100.00%	86.96%	85.56%
	1	1%	95.56%	87.50%	70.00%	77.78%	
	2	5%	96.11%	78.26%	90.00%	83.72%	
	3	10%	93.89%	71.43%	75.00%	73.17%	
	4	20%	94.44%	77.78%	70.00%	73.68%	
	5	40%	97.22%	100.00%	75.00%	85.71%	
	6	60%	99.44%	95.24%	100.00%	97.56%	
	7	80%	98.89%	90.91%	100.00%	95.24%	
	8	100%	98.89%	100.00%	90.00%	94.74%	

**Table 6:** Severity assessment results of the single wheat leaves with the actual percentages of lesion areas of all the severity classes of wheat stripe rust contained in the training and testing sets, obtained by using the severity assessment models of wheat stripe rust built based on spectral clustering under the condition of considering single healthy wheat leaves.

Dataset	Severity category	Severity class	Accuracy	Precision	Recall	F1 score	Overall accuracy
Train940	0	0%	99.44%	95.24%	100.00%	97.56%	96.94%
	1	1%	99.44%	100.00%	95.00%	97.44%	
	2	5%	98.33%	86.96%	100.00%	93.02%	
	3	10%	98.33%	100.00%	85.00%	91.89%	
	4	20%	100.00%	100.00%	100.00%	100.00%	
	5	40%	100.00%	100.00%	100.00%	100.00%	
	6	60%	100.00%	100.00%	100.00%	100.00%	
	7	80%	99.17%	93.02%	100.00%	96.39%	
	8	100%	99.17%	100.00%	92.50%	96.10%	
Test910	0	0%	98.89%	90.91%	100.00%	95.24%	97.78%
	1	1%	98.89%	100.00%	90.00%	94.74%	
	2	5%	98.89%	90.91%	100.00%	95.24%	
	3	10%	98.89%	100.00%	90.00%	94.74%	
	4	20%	100.00%	100.00%	100.00%	100.00%	
	5	40%	100.00%	100.00%	100.00%	100.00%	
	6	60%	100.00%	100.00%	100.00%	100.00%	
	7	80%	100.00%	100.00%	100.00%	100.00%	
	8	100%	100.00%	100.00%	100.00%	100.00%	
Train930	0	0%	99.26%	93.75%	100.00%	96.77%	96.67%
	1	1%	99.26%	100.00%	93.33%	96.55%	
	2	5%	98.52%	88.24%	100.00%	93.75%	
	3	10%	98.52%	100.00%	86.67%	92.86%	
	4	20%	100.00%	100.00%	100.00%	100.00%	
	5	40%	99.63%	100.00%	96.67%	98.31%	
	6	60%	99.63%	96.77%	100.00%	98.36%	
	7	80%	99.26%	93.75%	100.00%	96.77%	
	8	100%	99.26%	100.00%	93.33%	96.55%	
Test920	0	0%	99.44%	95.24%	100.00%	97.56%	95.56%
	1	1%	99.44%	100.00%	95.00%	97.44%	
	2	5%	98.33%	86.96%	100.00%	93.02%	
	3	10%	98.33%	100.00%	85.00%	91.89%	
	4	20%	99.44%	95.24%	100.00%	97.56%	
	5	40%	98.89%	100.00%	90.00%	94.74%	
	6	60%	99.44%	95.24%	100.00%	97.56%	
	7	80%	98.89%	90.91%	100.00%	95.24%	
	8	100%	98.89%	100.00%	90.00%	94.74%	

Under the condition of considering single healthy wheat leaves, by using the severity assessment model of wheat stripe rust built with the *K*-means clustering algorithm based on the training set Train940 when the modeling ratio was 4:1, the obtained assessment results of the training set Train940 as shown in **Table 5**, demonstrated that, for all the severity classes of the disease, high accuracies with the lowest value equal to 93.89% and the highest value equal to 99.17%, were achieved, but there were large differences in precision, recall, and F1 score. For all the severity classes of the disease in the training set Train940, the lowest and highest precisions were 71.43% and 100.00%, respectively; the lowest and highest recalls were 70.00% and 100.00%, respectively; and the lowest and highest F1 scores were 73.17% and 96.39%, respectively. The overall accuracy of the training set Train940 achieved by using the model was 84.72%. By using the built model to conduct the severity assessments of the specimens in the testing set Test910, for all the severity classes of wheat stripe rust, high accuracies were achieved, with the lowest value equal to 93.33% and the highest value equal to 100.00%; there were also large differences in precision, recall, and F1 score. The lowest precision, recall, and F1 score were all 70.00%, and the highest precision, recall, and F1 score were all 100.00%. For the built severity assessment model, the overall accuracy of the testing set Test910 was 85.56%.

As shown in **Table 6**, under the condition of considering single healthy wheat leaves, by using the severity assessment model of wheat stripe rust built with spectral clustering based on the training set Train940 when the modeling ratio was 4:1, the obtained assessment results for all the severity classes of wheat stripe rust in the training set Train940 showed that the lowest and highest accuracies was 98.33% and 100.00%, respectively; except that the precisions for the severity classes of 0%, 5%, and 80% were 95.24%, 86.96%, and 93.02%, respectively, the precisions for all other severity classes were 100.00%; except that the recalls for the severity classes of 1%, 10% and 100% were 95.00%, 85.00%, and 92.50%, respectively, the recalls for all other severity classes were 100.00%; and the lowest and highest F1 scores were 91.89% and 100.00%, respectively. By using the built model to conduct the severity assessments of the specimens in the testing set Test910, the obtained results showed that, except that the accuracies for the severity classes of 0%, 1%, 5%, and 10% were all 98.89%, the accuracies for other severity classes were all 100.00%; except that the precisions for the severity classes of 0% and 5% were both 90.91%, the precisions for other severity classes were all 100.00%; except that the recalls for the severity classes of 1% and 10% were both 90.00%, the recalls for other severity classes were all 100.00%; except that the F1 scores for the severity classes of 0%, 1%, 5%, and 10% were 95.24%, 94.74%, 95.24%, and 94.74%, respectively, the F1 scores for all other severity classes were 100.00%. For the severity assessment model of wheat stripe rust built with spectral clustering based on the training set Train940, the overall accuracies of the training set Train940 and the corresponding testing set Test910 were 96.94% and 97.78%, respectively. The results indicated that very good severity assessment results for the specimens in both the training set and the testing set were achieved by using the severity assessment model built with spectral clustering when the modeling ratio was 4:1 under the condition of considering single healthy wheat leaves.

Under the condition of considering single healthy wheat leaves, when the modeling ratio was 3:2, by using the severity assessment model of wheat stripe rust built with the *K*-means clustering algorithm based on the training set Train930, the severity assessment results (**Table 5**) showed that, for all the severity classes of wheat stripe rust in the training set Train930, high accuracies were obtained, with the lowest value equal to 93.70% and the highest value equal to 99.26%; the lowest and highest precisions were 70.97% and 100.00%, respectively; the lowest and highest recalls were 70.00% and 100.00%, respectively; and the lowest and highest F1 scores were 72.13% and 96.77%, respectively. The overall accuracy of the training set Train930 achieved by using the built severity assessment model was 85.19%. The results indicated that there were great differences in precisions, recalls, and F1 scores for all the severity classes in the training set Train930 achieved by using the model built based on the *K*-means clustering algorithm. The assessment results obtained by using the severity assessment model of wheat stripe rust built with the *K*-means clustering algorithm based on the training set Train930 showed that, for all the severity classes of wheat stripe rust in the testing set Train920, the lowest and highest accuracies were 93.89% and 99.44%, respectively; the lowest and highest precisions were 71.43% and 100.00%, respectively; the lowest and highest recalls were 70.00% and 100.00%, respectively; and the lowest and highest F1 scores were 73.17% and 97.56%, respectively. There were also great differences in precisions, recalls, and F1 scores for all the severity classes in the testing set Train920 achieved by using the model built based on the *K*-means clustering algorithm under the condition of considering single healthy wheat leaves. For the built severity assessment model, the overall accuracy of the testing set Test920 was 85.56%.

As shown in **Table 6**, under the condition of considering single healthy wheat leaves, by using the severity assessment model of wheat stripe rust built with spectral clustering based on the training set Train930 when the modeling ratio was 3:2, the obtained assessment results showed that, for all the severity classes of wheat stripe rust in the training set Train930, the lowest and highest accuracies were 98.52% and 100.00%, respectively; the lowest and highest precisions were 88.24% and 100.00%, respectively; except that the recalls for the severity classes of 1%, 10%, 40%, and 100% were 93.33%, 86.67%, 96.67%, and 93.33%, respectively, the recalls for other severity classes were all 100.00%; and the lowest F1 score was 92.86% and the highest F1 score was 100.00%. The assessment results obtained by using the severity assessment model of wheat stripe rust built with spectral clustering based on the training set Train930 showed that, for all the severity classes of wheat stripe rust in the testing set Test920, the lowest and highest accuracies were 98.33% and 99.44%, respectively; the lowest and highest precisions were 86.96% and 100.00%, respectively; except that the recalls for the severity classes of 1%, 10%, 40%, and 100% were 95.00%, 85.00%, 90.00%, and 90.00%, respectively, the recalls for all other severity classes were 100.00%; and the lowest F1 score was 91.89%, and the highest F1 score was 97.56%. For the severity assessment model of wheat stripe rust built with spectral clustering based on Train930, the overall accuracies of the training set Train930 and the corresponding testing set Test920 were 96.67% and 95.56%, respectively. The results indicated that, under the condition of considering single healthy wheat leaves, very good severity assessment performance on both the training set Train930 and the corresponding testing set Test920 were achieved by using the severity assessment model of wheat stripe rust built with spectral clustering.

The results showed that, when the severity assessments of the single wheat leaf image datasets containing single healthy wheat leaves were conducted by using the severity assessment models, built based on the two unsupervised learning methods including the *K*-means clustering algorithm and spectral clustering, respectively, the achieved assessment performances were acceptable. When the modeling ratio was 4:1 or 3:2, the assessment performance obtained by using the severity assessment model of wheat stripe rust based on spectral clustering was better than that obtained by using the severity assessment model of wheat stripe rust based on the *K*-means clustering algorithm, and very ideal assessment performances on both the training set and the corresponding testing set could be achieved by using the severity assessment model of wheat stripe rust based on spectral clustering, indicating that the severity assessment model of wheat stripe rust based on spectral clustering could be treated as the optimal model for carrying out severity assessment of wheat stripe rust. Therefore, to achieve ideal severity assessment results by using the severity assessment method of wheat stripe rust based on unsupervised learning under the condition of considering single healthy wheat leaves, the severity assessment model of the disease can be built based on spectral clustering. The results demonstrated that the methods for severity assessment of wheat stripe rust based on unsupervised learning proposed in this study could be utilized to carry out severity assessment of wheat stripe rust under the condition of considering single healthy wheat leaves.

### 3.5 Severity assessment results obtained by using the severity assessment models of wheat stripe rust built based on the three supervised learning methods including the SVM, RF, and KNN under the condition of considering single healthy wheat leaves

Under the condition of considering single healthy wheat leaves, when the modeling ratio was 4:1, based on the training set Train940, the optimal SVM model for severity assessment of wheat stripe rust was built with the optimal parameter *C* of 2.297 and the optimal parameter *g* of 4.000. By using this SVM model to carry out the severity assessments of the specimens in the training set Train940 and the testing set Test910, for the severity class of 0% (healthy wheat leaves) in the training set Train940, the accuracy, precision, recall, and F1 score were 99.72%, 97.56%, 100.00%, and 98.77%, respectively; for the severity class of 1% in the training set Train940, the accuracy, precision, recall, and F1 score were 99.72%, 100.00%, 97.50%, and 98.73%, respectively; and for all other severity classes in the training set Train940 and all the severity classes in the testing set Test910, the accuracies, precisions, recalls, and F1 scores were all 100.00%. For the built optimal SVM model for severity assessment of wheat stripe rust when the modeling ratio was 4:1 under the condition of considering single healthy wheat leaves, the overall accuracies of the training set Train940 and the corresponding testing set Test910 were 99.72% and 100.00%, respectively (**Table 7**).

**Table 7:** Overall accuracies of the training and testing sets obtained by using the built severity assessment SVM, RF, and KNN models of wheat stripe rust under the condition of considering single healthy wheat leaves.

Modeling method	Modeling ratio	Overall accuracy of the training set	Overall accuracy of the testing set
SVM	4:1	99.72%	100.00%
	3:2	99.63%	100.00%
RF	4:1	100.00%	100.00%
	3:2	100.00%	100.00%
KNN	4:1	99.17%	100.00%
	3:2	99.63%	100.00%

The table shows only the assessment results of the optimal severity assessment SVM, RF, and KNN models of wheat stripe rust.

Under the condition of considering single healthy wheat leaves, when the modeling ratio was 3:2, based on the training set Train930, the optimal SVM model for severity assessment of wheat stripe rust was built with the optimal parameter *C* equal to 194.012 and the optimal parameter *g* equal to 0.330. By using this SVM model to perform the severity assessments of the specimens in the training set Train930 that was used for modeling and the corresponding testing set Test920, for the severity class of 1% in the training set Train930, the accuracy, precision, recall, and F1 score were 99.63%, 100.00%, 96.67%, and 98.31%, respectively; for the severity class of 5% in the training set Train930, the accuracy, precision, recall, and F1 score were 99.63%, 96.77%, 100.00%, and 98.36%, respectively; and for all other severity classes in the training set Train930 and all the severity classes in the testing set Test920, the accuracies, precisions, recalls, and F1 scores were all 100.00%. For the built optimal SVM model for severity assessment of wheat stripe rust when the modeling ratio was 3:2 under the condition of considering single healthy wheat leaves, the overall accuracies of the training set Train930 and the corresponding testing set Test920 were 99.63% and 100.00%, respectively (**Table 7**).

Under the condition of considering single healthy wheat leaves, when the modeling ratio was 4:1, based on the training set Train940, the optimal RF model for severity assessment of wheat stripe rust was built with the optimal number of decision trees equal to 10; and when the modeling ratio was 3:2, based on the training set Train930, the optimal RF model for severity assessment of wheat stripe rust was built with the optimal number of decision trees equal to 20. Under the two conditions of the modeling ratios equal to 4:1 and 3:2, the optimal RF models built for severity assessment of wheat stripe rust were used to conduct the severity assessments of the specimens in the training sets (Train940 and Train930) and the testing sets (Test910 and Test920), and for all the severity classes of wheat stripe rust in the training sets and the testing sets, the accuracies, precisions, recalls, and F1 scores were all 100.00%. For each modeling ratio under the condition of considering single healthy wheat leaves, by using the optimal RF model built for severity assessment of wheat stripe rust, the overall accuracies of the training set that was used for modeling and the corresponding testing set were both 100.00% (**Table 7**).

Under the condition of considering single healthy wheat leaves, when the modeling ratio was 4:1, based on the training set Train940, the optimal KNN model for severity assessment of wheat stripe rust was built with the optimal *K* of 9. By using this KNN model to perform the severity assessments of the specimens in the training set Train940 and the testing set Test910, for the severity classes of 0%, 5%, and 80% in the training set Train940, the accuracies were all 99.72%, the precision were all 97.56%, the recall were all 100.00%, and F1 score were all 98.77%; for the severity classes of 1%, 10%, and 100% in the training set Train940, the accuracies were all 99.72%, the precisions were all 100.00%, the recalls were all 97.50%, and the F1 scores were all 98.73%; and for all other severity classes in the training set Train940 and all the severity classes in the testing set Test910, all the accuracies, precisions, recalls, and F1 scores were 100.00%. As shown in **Table 7**, for the built optimal KNN model for severity assessment of wheat stripe rust when the modeling ratio was 4:1 under the condition of considering single healthy wheat leaves, the overall accuracy of the training set Train940 was 99.17%, and that of the corresponding testing set Test910 was 100.00%.

Under the condition of considering single healthy wheat leaves, when the modeling ratio was 3:2, based on the training set Train930, the optimal KNN model for severity assessment of wheat stripe rust was built with the optimal *K* equal to 5. By using this optimal KNN model to perform the severity assessments of the specimens in the training set Train930 and the testing set Test920, for the severity class of 0% in the training set Train930, the accuracy, precision, recall, and F1 score were 99.63%, 96.77%, 100.00%, and 98.36%, respectively; for the severity class of 1% in the training set Train930, the accuracy, precision, recall, and F1 score were 99.63%, 100.00%, 96.67%, and 98.31%, respectively; and for all other severity classes in the training set Train930 and all the severity classes in the testing set Test920, all the accuracies, precisions, recalls, and F1 scores were 100.00%. As shown in **Table 7**, for the built optimal KNN model for severity assessment of wheat stripe rust when the modeling ratio was 3:2 under the condition of considering single healthy wheat leaves, the overall accuracies of the training set Train930 and the corresponding testing set Test920 were 99.63% and 100.00%, respectively.

The results indicated that, under the condition of considering single healthy wheat leaves, very good severity assessment performances on the training sets (Train940 and Train930) and testing sets (Test910 and Test920) could be obtained by using the built optimal SVM, RF, and KNN models for severity assessment of wheat stripe rust. Furthermore, in the case of the two modeling ratios of 4:1 and 3:2, the severity assessment performances of the built optimal RF models were the best, and the overall accuracies of the training sets and the testing sets were all 100.00%. The obtained results indicated that the severity assessment methods based on supervised learning for wheat stripe rust proposed in this study could be used to carry out severity assessment of wheat stripe rust under the condition of considering single healthy wheat leaves.

## 4 Discussion

In this study, the methods for severity assessment of wheat stripe rust were proposed based on machine learning. Regardless of whether the healthy wheat leaves were considered or not, acceptable assessment performances could be obtained by using the severity assessment models of wheat stripe rust, built with the two unsupervised learning methods including the *K*-means clustering algorithm and spectral clustering, based on the training sets and the testing sets constructed by using the system sampling method with the modeling ratios of 4:1 and 3:2. Especially, high accuracies, precisions, recalls, F1 scores, and overall accuracies were obtained on both the training sets and the testing sets by using the severity assessment models of wheat stripe rust built based on spectral clustering, and the relatively ideal performances for severity assessments of wheat stripe rust were achieved. Regardless of whether the healthy wheat leaves were considered or not, very good assessment results were achieved by using the severity assessment models of wheat stripe rust built based on the three supervised learning methods including SVM, RF, and KNN when the modeling ratio were 4:1 and 3:2. In particular, the accuracies, precisions, recalls, and F1 scores for all the severity classes of the training and testing sets and the overall accuracies of the training and testing sets, were all 100.00%, by using the optimal models (the optimal RF models) for severity assessment of wheat stripe rust obtained by comparing the three modeling methods of SVM, RF and KNN, and thus, very ideal performances for severity assessments of wheat stripe rust were achieved by using the selected optimal models (the optimal RF models). The results indicated that the methods for wheat stripe rust severity assessment based on unsupervised learning and supervised learning proposed in this study could be used for severity assessment of wheat stripe rust. For the proposed severity assessment methods of wheat stripe rust in this study, the model building and severity assessments were conducted based on the actual percentages of lesion areas in the areas of the corresponding whole single diseased wheat leaves. The problem that, when severity assessment of wheat stripe rust is carried out based on the percentage of the lesion area in the area of a whole single diseased wheat leaf, the percentage of the lesion area in the area of the whole single diseased wheat leaf corresponding to a severity class in the severity grading standard of the disease is not inconsistent with the actual percentage of the lesion area in the area of the whole single diseased wheat leaf, was completely solved in this study. By using the methods proposed in this study, based on the actual percentage of the lesion area in the whole area of a single diseased wheat leaf, the severity of the corresponding diseased leaf can be directly assessed. The results obtained in this study provide a basis for accurately assessing the severity of wheat stripe rust. The methods and ideas provided in this study are also applicable to other plant diseases such as wheat leaf rust caused by *Puccinia triticina*, for which the percentage of the lesion area in the corresponding diseased plant unit area of a severity class in the corresponding disease severity grading standard is not inconsistent with the actual percentage of the lesion area in the area of the whole diseased plant unit. They are also applicable to other plant diseases for which disease severity assessment of a diseased plant unit is carried out based on the ratio of the lesion area to the area of the whole diseased plant unit. They can be used to solve such problems in the severity assessments of plant diseases. In this study, two unsupervised learning methods including the *K*-means clustering algorithm and spectral clustering and three supervised learning methods including SVM, RF, and KNN were used to build the severity assessment models of wheat stripe rust, respectively. By using the ideas provided in this study, other unsupervised learning methods and supervised learning methods can be used to build the severity assessment models of wheat stripe rust in further studies.

The severity assessment methods of wheat stripe rust proposed in this study were to build the severity assessment models of wheat stripe rust based on the actual percentages of lesion areas in the areas of the corresponding whole single diseased wheat leaves by using the unsupervised learning methods and the supervised learning methods. Then, by using the built severity assessment models of wheat stripe rust, the severity classes of the single diseased wheat leaves with the actual percentages of lesion areas could be directly assessed. In particular, when an unsupervised learning method is used to build the severity assessment model of wheat stripe rust, there is no need to artificially determine the severity classes of the single diseased wheat leaves with the actual percentages of lesion areas, the single diseased wheat leaves can be classified and assessed by using the unsupervised learning method according to the number of severity classes in the disease severity grading standard, and then, through optimization of the built severity assessment model, a model for severity assessment of wheat stripe rust with satisfactory assessment performance can be obtained. Unsupervised learning is conducive to the application of the developed severity assessment methods in practice, and can reduce the errors in severity assessments caused by using the visual observation method. The severity assessment methods of wheat stripe rust based on machine learning developed in this study has strong practical applicability and can realize the accurate severity assessment of the disease, which is of great significance for the survey, monitoring, prediction, and control of the disease. After the severity classes of wheat leaves are assessed, disease prevalence evaluation and disease predication can be performed, and then suitable control measures can be made. Generally, the evaluated disease prevalence data or the disease prediction results are compared to the control threshold or economic threshold of the disease to determine whether the disease needs to be controlled. Once the control threshold or economic threshold is reached, suitable measures, such as spraying fungicides, can be taken to control the disease.

At the present time, severity assessments of wheat stripe rust based on image processing technology are mainly realized by comparing the actual percentage of the lesion area in the area of a whole single diseased wheat leaf obtained by using image processing to the percentages for the eight severity classes in the severity grading standard of the disease (Jiang et al., 2021) or by building the severity assessment models of wheat stripe rust based on extracted features of disease images (Bao et al., 2021). The percentage of the lesion area in the area of a whole diseased wheat leaf corresponding to each severity class in the severity grading standard of wheat stripe rust is obviously greater than the actual percentage of the lesion area in the area of the whole diseased wheat leaf, which may cause great errors in the disease severity assessments. Jiang et al. (2022) determined the reference ranges for severity assessment of wheat stripe rust based on the actual percentages of lesion areas corresponding to each severity class of wheat stripe rust, and then obtained satisfactory assessment results (the severity assessment accuracies for the training sets and testing sets were not lower than 85%.) of the diseased wheat leaves with the actual percentages of lesion areas according to the determined reference ranges. On the basis of the study conducted by Jiang et al. (2022), the severity assessment models of wheat stripe rust were built based on machine learning with the actual percentages of the lesion areas in the areas of the corresponding single leaves of wheat stripe rust and the corresponding severity category data in this study. According to the calculating methods, the two indicators used to evaluate the performances of severity assessment methods, recall used in this study and severity assessment accuracy used by Jiang et al. (2022), are the same. In terms of recall used in this study and severity assessment accuracy used by Jiang et al. (2022), a comparison of the performances of the methods developed in this study to those of the methods developed by Jiang et al. (2022) was made, and the results showed that the performance of the severity assessment method based on the *K*-means clustering algorithm developed in this study was the worst and that of the severity assessment method based on RF developed in this study was the best (**Tables 8**, **9**). The severity assessment methods of wheat stripe rust based on machine learning developed in this study will greatly improve the accuracy of image-based severity assessment of the disease, and can greatly reduce the requirements for the experience of assessors/raters in disease severity assessment. This is beneficial to improve the reliability of the monitoring and early warning information of wheat stripe rust, and is conducive to the popularization and application of related technologies. This will be helpful to improve the level of the survey, monitoring and early warning, and management of wheat stripe rust. The automatization and intellectualization of plant disease severity assessment is an inevitable development trend of the science and technology (Wang, 2022). This study provides ideas and basis for accurate severity assessment of wheat stripe rust based on image processing technology, which is conducive to the development of automatic severity assessment system of the disease and the improvement of the severity assessment level of the disease. This is useful to promoting the automatization and intellectualization of severity assessment of wheat stripe rust, and can provide more reliable support for the prediction, variety resistance identification and variety breeding, and disease control strategy making of wheat stripe rust.

**Table 8:** Comparison results of the performances of the machine-learning-based methods developed in this study to those of the reference-range-based methods developed by Jiang et al. (2022) when the modeling/sampling ratio was 4:1, in terms of recall used in this study and severity assessment accuracy used by Jiang et al. (2022) that are the same according to their calculating methods.

Dataset	Severity class	Recall used in this study										Severity assessment accuracy used by Jiang et al. (2022)			
		*K*-means clustering algorithm	Spectral clustering	SVM	RF	KNN	*K*-means clustering algorithm	Spectral clustering	SVM	RF	KNN	The midpoint-of-two-adjacent-means-based actual percentage reference range	The 90% reference range	The 95% reference range	The 99% reference range
Training set	0%	–	–	–	–	–	100.00%	100.00%	100.00%	100.00%	100.00%	–	–	–	–
	1%	100.00%	100.00%	100.00%	100.00%	97.50%	70.00%	95.00%	97.50%	100.00%	97.50%	100.00%	95.00%	97.50%	100.00%
	5%	95.00%	100.00%	100.00%	100.00%	100.00%	85.00%	100.00%	100.00%	100.00%	100.00%	100.00%	95.00%	100.00%	100.00%
	10%	75.00%	87.50%	100.00%	100.00%	100.00%	75.00%	85.00%	100.00%	100.00%	97.50%	87.50%	90.00%	100.00%	100.00%
	20%	70.00%	100.00%	100.00%	100.00%	100.00%	70.00%	100.00%	100.00%	100.00%	100.00%	97.50%	95.00%	100.00%	100.00%
	40%	75.00%	100.00%	100.00%	100.00%	100.00%	75.00%	100.00%	100.00%	100.00%	100.00%	85.00%	95.00%	95.00%	100.00%
	60%	100.00%	100.00%	100.00%	100.00%	100.00%	100.00%	100.00%	100.00%	100.00%	100.00%	100.00%	92.50%	97.50%	95.00%
	80%	97.50%	100.00%	100.00%	100.00%	100.00%	97.50%	100.00%	100.00%	100.00%	100.00%	97.50%	90.00%	97.50%	100.00%
	100%	90.00%	92.50%	100.00%	100.00%	100.00%	90.00%	92.50%	100.00%	100.00%	97.50%	90.00%	87.50%	100.00%	100.00%
Testing set	0%	–	–	–	–	–	100.00%	100.00%	100.00%	100.00%	100.00%	–	–	–	–
	1%	100.00%	100.00%	100.00%	100.00%	100.00%	70.00%	90.00%	100.00%	100.00%	100.00%	100.00%	90.00%	100.00%	100.00%
	5%	90.00%	100.00%	100.00%	100.00%	100.00%	90.00%	100.00%	100.00%	100.00%	100.00%	100.00%	90.00%	100.00%	100.00%
	10%	70.00%	90.00%	100.00%	100.00%	100.00%	70.00%	90.00%	100.00%	100.00%	100.00%	90.00%	90.00%	100.00%	100.00%
	20%	70.00%	100.00%	100.00%	100.00%	100.00%	70.00%	100.00%	100.00%	100.00%	100.00%	100.00%	100.00%	100.00%	100.00%
	40%	80.00%	100.00%	100.00%	100.00%	100.00%	80.00%	100.00%	100.00%	100.00%	100.00%	90.00%	100.00%	100.00%	100.00%
	60%	100.00%	100.00%	100.00%	100.00%	100.00%	100.00%	100.00%	100.00%	100.00%	100.00%	100.00%	90.00%	100.00%	100.00%
	80%	100.00%	100.00%	100.00%	100.00%	100.00%	100.00%	100.00%	100.00%	100.00%	100.00%	100.00%	90.00%	100.00%	100.00%
	100%	90.00%	100.00%	100.00%	100.00%	100.00%	90.00%	100.00%	100.00%	100.00%	100.00%	90.00%	90.00%	100.00%	100.00%

‘–’ in the table denotes that single healthy wheat leaves were not considered.

**Table 9:** Comparison results of the performances of the machine-learning-based methods developed in this study to those of the reference-range-based methods developed by Jiang et al. (2022) when the modeling/sampling ratio was 3:2, in terms of recall used in this study and severity assessment accuracy used by Jiang et al. (2022) that are the same according to their calculating methods.

Dataset	Severity class	Recall used in this study										Severity assessment accuracy used by Jiang et al. (2022)			
		*K*-means clustering algorithm	Spectral clustering	SVM	RF	KNN	*K*-means clustering algorithm	Spectral clustering	SVM	RF	KNN	The midpoint-of-two-adjacent-means-based actual percentage reference range	The 90% reference range	The 95% reference range	The 99% reference range
Training set	0%	–	–	–	–	–	100.00%	100.00%	100.00%	100.00%	100.00%	–	–	–	–
	1%	100.00%	100.00%	100.00%	100.00%	100.00%	70.00%	93.33%	96.67%	100.00%	96.67%	100.00%	93.33%	96.67%	100.00%
	5%	93.33%	100.00%	100.00%	100.00%	100.00%	90.00%	100.00%	100.00%	100.00%	100.00%	100.00%	93.33%	100.00%	100.00%
	10%	73.33%	90.00%	100.00%	100.00%	100.00%	73.33%	86.67%	100.00%	100.00%	100.00%	86.67%	90.00%	96.67%	96.67%
	20%	66.67%	100.00%	100.00%	100.00%	100.00%	70.00%	100.00%	100.00%	100.00%	100.00%	96.67%	96.67%	100.00%	100.00%
	40%	76.67%	100.00%	100.00%	100.00%	100.00%	76.67%	96.67%	100.00%	100.00%	100.00%	86.67%	96.67%	96.67%	96.67%
	60%	100.00%	100.00%	100.00%	100.00%	100.00%	100.00%	100.00%	100.00%	100.00%	100.00%	100.00%	93.33%	96.67%	100.00%
	80%	76.67%	100.00%	100.00%	100.00%	100.00%	96.67%	100.00%	100.00%	100.00%	100.00%	96.67%	93.33%	96.67%	100.00%
	100%	83.33%	90.00%	100.00%	100.00%	100.00%	90.00%	93.33%	100.00%	100.00%	100.00%	90.00%	90.00%	100.00%	100.00%
Testing set	0%	–	–	–	–	–	100.00%	100.00%	100.00%	100.00%	100.00%	–	–	–	–
	1%	100.00%	100.00%	100.00%	100.00%	100.00%	70.00%	95.00%	100.00%	100.00%	100.00%	100.00%	95.00%	100.00%	100.00%
	5%	90.00%	100.00%	100.00%	100.00%	100.00%	90.00%	100.00%	100.00%	100.00%	100.00%	100.00%	95.00%	100.00%	100.00%
	10%	70.00%	85.00%	100.00%	100.00%	100.00%	75.00%	85.00%	100.00%	100.00%	100.00%	90.00%	90.00%	95.00%	95.00%
	20%	70.00%	100.00%	100.00%	100.00%	100.00%	70.00%	100.00%	100.00%	100.00%	100.00%	100.00%	95.00%	100.00%	100.00%
	40%	75.00%	95.00%	100.00%	100.00%	100.00%	75.00%	90.00%	100.00%	100.00%	100.00%	85.00%	95.00%	95.00%	100.00%
	60%	100.00%	100.00%	100.00%	100.00%	100.00%	100.00%	100.00%	100.00%	100.00%	100.00%	100.00%	90.00%	100.00%	100.00%
	80%	80.00%	100.00%	100.00%	100.00%	100.00%	100.00%	100.00%	100.00%	100.00%	100.00%	100.00%	90.00%	100.00%	100.00%
	100%	85.00%	90.00%	100.00%	100.00%	100.00%	90.00%	90.00%	100.00%	100.00%	100.00%	90.00%	85.00%	100.00%	100.00%

‘–’ in the table denotes that single healthy wheat leaves were not considered.

In this study, the actual percentages of lesion areas in the areas of the corresponding whole single diseased leaves infected by wheat stripe rust were obtained by using image processing technology with image processing software. The actual percentage data can be obtained by assessors/raters using the visual observation method. In addition, the actual percentages of lesion areas can be obtained by programming to implement the segmentation of lesion images and the calculation of ratios of the lesion areas to the areas of the corresponding whole single diseased leaves (Li et al., 2011; Jiang et al., 2021), by using special software and packages (Lamari, 2008; Schneider et al., 2012; Pethybridge and Nelson, 2015; Olivoto et al., 2022), and by actual experimental operation methods such as graph paper method and paper-weighing method (Li et al., 2011). The automatic lesion segmentation methods and the automatic calculation methods of the actual percentages of lesion areas in the areas of the corresponding whole single diseased leaves can be combined with the severity assessment methods and models developed in this study to construct automatic severity assessment systems of wheat stripe rust, which is conducive to the implementation of automatic severity assessment of wheat stripe rust and the more convenient practical applications of related technical methods.

By using the reference-range-based methods proposed by Jiang et al. (2022) to assess the severity class of a single diseased wheat leaf infected with wheat stripe rust, the actual percentage of the lesion area in the area of the whole single diseased leaf needs to be compared to the upper and lower limits of the reference ranges of all the severity classes of the disease. For the severity assessment methods of wheat stripe rust based on machine learning proposed in this study, the actual percentage of the lesion area in the area of a whole single diseased wheat leaf infected with wheat stripe rust needs to be input into the built severity assessment models, and then, the severity class of the single diseased wheat leaf to be assessed can be determined. The severity assessment methods of wheat stripe rust proposed in this study are different from those proposed by Jiang et al. (2022), however, by using all these methods, satisfactory assessment results can be achieved. Both the severity assessment methods developed in this study and those proposed by Jiang et al. (2022) can be automated by computer programming. According to the actual situation, these methods can be integrated with disease image processing systems, and the severity assessment functions of these systems can be improved or the severity assessment functions can be added to these systems, to realize the automation of severity assessment of wheat stripe rust.

In the surveys and assessments of wheat stripe rust, severity and infection type are two different terms. Generally, infection type is used as an important indicator to evaluate the resistance of wheat to stripe rust. The same strain or physiological race of *Pst* can cause different infection types on different wheat varieties. Determination of infection type is a kind of qualitative evaluation, which can be implemented by using extracted image features to identify the categories of infection types based on image processing technology (Hayit et al., 2021). Different severity classes of wheat stripe rust can also be qualitatively identified based on image processing technology (Bao et al., 2021). In fact, the actual percentage of lesion area is a continuous variable. Therefore, the disease severity assessment method based on the actual percentages of the lesion areas should be the best solution to implement severity assessment of wheat stripe rust.

## 5 Conclusions

In this study, efforts were made to solve the problems existing in the severity assessment methods of wheat stripe rust and to develop new methods for severity assessment of the disease based on machine learning. The acquired actual percentage data of the lesion areas of single diseased wheat leaves were used to construct the training sets and the corresponding testing sets by using the system sample method with two modeling ratios under the two conditions of considering healthy single wheat leaves or not, and then, the two unsupervised learning methods including *K*-means clustering algorithm and spectral clustering and the three supervised learning methods including SVM, RF, and KNN were used to build the severity assessment models of wheat stripe rust, respectively. By using the built models to carry out the severity assessments of the training and testing sets, satisfactory assessment results were achieved. In particular, the severity classes of all the specimens in the training and testing sets can correctly assessed by using the built optimal RF models for severity assessment of wheat stripe rust. The results indicated that good assessment performance can be achieved by using the disease severity assessment methods developed in this study, and that the methods are suitable for the severity assessment of wheat stripe rust. The severity assessment methods of wheat stripe rust based on machine learning were provided in this study. For the methods, the obtained actual percentages of lesion areas in the areas of the whole single diseased wheat leaves were directly used to build the severity assessment models of wheat stripe rust, and then, the built models were used to conduct severity assessments of single wheat leaves based on the obtained corresponding actual percentages of lesion areas of the leaves. The methods are simple, rapid, and easy-to-operate, and by using these methods, very highly accurate assessment results of single wheat leaves can be achieved. More importantly, the severity assessment methods proposed in this study can provide a reference for the severity assessments of all the plant diseases for which the severity assessments are performed based on the actual ratios of lesion areas to the areas of the diseased plant units, and this study can provide a basis for the implementation of automatic severity assessments of plant diseases by computer programming based on computer vision technology and image processing technology. In future studies, automatic severity assessment systems of plant diseases can be developed based on the proposed severity assessment methods. It is feasible for UAV (unmanned aerial vehicle) applications in field environments under the conditions that actual ratios of lesion areas of plant units could be obtained.

## Data availability statement

The original contributions presented in the study are included in the article/supplementary material. Further inquiries can be directed to the corresponding author. 

## Author contributions

HGW contributed conception of the study and designed the experiments. QJ, HLW and HGW performed the experiments; QJ, HLW and HGW analyzed the data; QJ and HGW wrote the draft of the manuscript. All authors contributed to the article and approved the submitted version.
